# Comparison of the Apoptotic Effects of Supercritical Fluid Extracts of *Antrodia cinnamomea* Mycelia on Hepatocellular Carcinoma Cells

**DOI:** 10.3390/molecules19079033

**Published:** 2014-06-27

**Authors:** Hsiu-Man Lien, Chun-Hung Chiu, Chia-Chang Chen, Wan-Lin Chang, Charng-Cherng Chyau, Robert Y. Peng

**Affiliations:** 1 Department of Chemistry, Tunghai University, No.1727, Sec. 4, Taiwan Boulevard, Xitun District, Taichung 40704, Taiwan; 2 Research Institute of Biotechnology, Hungkuang University, No. 1018, Sec. 6, Taiwan Boulevard, Shalu District, Taichung 43302, Taiwan; 3 Yusheng Biotechnology Center, No. 26, Keyuan Rd., Xitun District, Taichung 40763, Taiwan

**Keywords:** *Antrodia cinnamomea*, solid-state culture, supercritical fluid extract, HepG2 cell, apoptotic activity

## Abstract

*Antrodia cinnamomea* (AC) has been widely used as a folk medicine in the prevention and treatment of liver diseases, such as hepatitis, hepatic fibrosis, and hepatocellular carcinoma. Previous studies have indicated that triterpenoids and benzenoids show selective cytotoxicity against human hepatoma cell lines. The aim of the study was to compare the triterpenoid content of extract and the extract-induced cytotoxicity in HepG2 cells from mycelia extracts of solid state cultured AC obtained by supercritical fluid extraction (SFE) and the conventional solvent extraction method. SFE with CO_2_ mixed with a constant amount of ethanol co-solvent (10% of CO_2_ volume) applied at different temperatures and pressures (40, 60 and 80 °C and, 20.7, 27.6 and 34.5 Mpa) was also compared in the study. Although the extraction yield of triterpenoids (59.7 mg/g) under the optimal extraction conditions of 34.5 MPa (5000 psi)/60 °C (designated as sample S-5000-60) was equivalent to the extraction yield using conventional liquid solvent extraction with ethanol (ETOH-E) at room temperature (60.33 mg/g), the cytotoxicity of the former against the proliferation of HepG2 cell line measured as the inhibition of 50% of cell growth activity (IC_50_) at dosages of 116.15, 57.82 and 43.96 µg/mL was superior to that of EtOH-E at 131.09, 80.04 and 48.30 µg/mL at 24, 48 and 72 h, respectively. Additionally, we further proved that the apoptotic effect of S-5000-60 presented a higher apoptosis ratio (21.5%) than ETOH-E (10.5%) according to annexin V-FITC and propidium iodide double staining assay results. The high affinity and selectivity of SFE on bioactive components resulted in a higher extraction efficiency than conventional solvent extraction. The chemical profile of the obtained extracts from solid state cultivated mycelium of AC was also determined by high-performance liquid chromatography electrospray ionization tandem mass spectrometry (LC-MS/MS), whereby three benzenoids and four triterpenoids were found for the first time in SFE extracts with 4,7-dimethoxy-5-methyl-l,3-benzodioxole (5.78 mg/g) being the most abundant component, followed by 2,4-dimethoxy-6-methylbenzene-1,3-diol (3.03 mg/g) and dehydroeburicoic acid (0.40 mg/g).

## 1. Introduction

*Antrodia cinnamomea* (AC, syn. *Antrodia camphorata*, *Taiwanofungus camphoratus and Ganoderma comphoratum*) is a new basidiomycete native only to Taiwan and originally found growing in the empty rotting trunk of *Cinnamomum kanehirai* Hay [1]. AC has been reported to have anti-inflammatory, hepatoprotective anti-fatigue, antitumor, antioxidant, and immunomodulatory effects [2,3,4,5,6,7,8]. However, because of its parasitic exclusively on the rot wood of the endemic species *Cinnamomum kanehirai*, the wild type fruiting body AC is being in danger of extinction [9]. Due to the limited distribution of the host plant and the slow growth rate of the fungus, in addition to submerged liquid culture, the use of solid-state culture systems has also been attempted for the mass production of the fungus [10].

The extracts from metabolites of solid-state culture have indicated protective activity against ethanol-induced oxidative stress in liver cells. Many bioactive components have been found in AC, such as triterpenoids, polysaccharides, benzenoids, benzoquinone derivatives, and succinic and maleic acid derivatives [11,12]. In fruiting bodies of AC, the rich amount of triterpenoids has been demonstrated to be the source of many biological activities, including immune-enhancing responses [13] and the liver-protective effects against chronic alcohol consumption damage [14]. Triterpenoids, being the major secondary metabolites in fruiting bodies of AC, offer an extraordinary source of antioxidant, anti-inflammation and anticancer biological activities [6,7,8]. However, the solid culture time and environment is very different from that wild growing fruiting body which has grown on *C. kanehirai* rotten trunks. Although solid culture of AC might be a plausible economic solution for replacing the wild growing sources, there are no reports regarding the chemical composition and antitumor effects of the solid culture AC products.

The SFE method has been reported do display some noteworthy advantages over traditional techniques, such as shorter extraction times, low environmental impact, and a clean, non-thermally-degraded final product of interest [15]. In the present study, we first report a comparison of the extractability of tritepenoids and benzenoids from solid state cultured mycelia of AC using the SFE and ethanol solvent extraction methods, indicating the most effective method for obtaining extracts with high anti-proliferation activity and also capable of inducing apoptosis activity on HepG2 cells. Moreover, to further understand the bioactive components from the AC extracts, we investigated the tritepenoid and benzenoid contents of the extracts by HPLC-UV and characterized the compounds using LC-ESI-MS-MS. The results implied that the AC triterpenoids and benzenoids possess potential anti-hepatoma activities and they exert their inhibition effects via apoptosis-inducing properties.

## 2. Results and Discussion

### 2.1. Yield and Triterpenoid Content of AC Extracts

**Figure 1:**
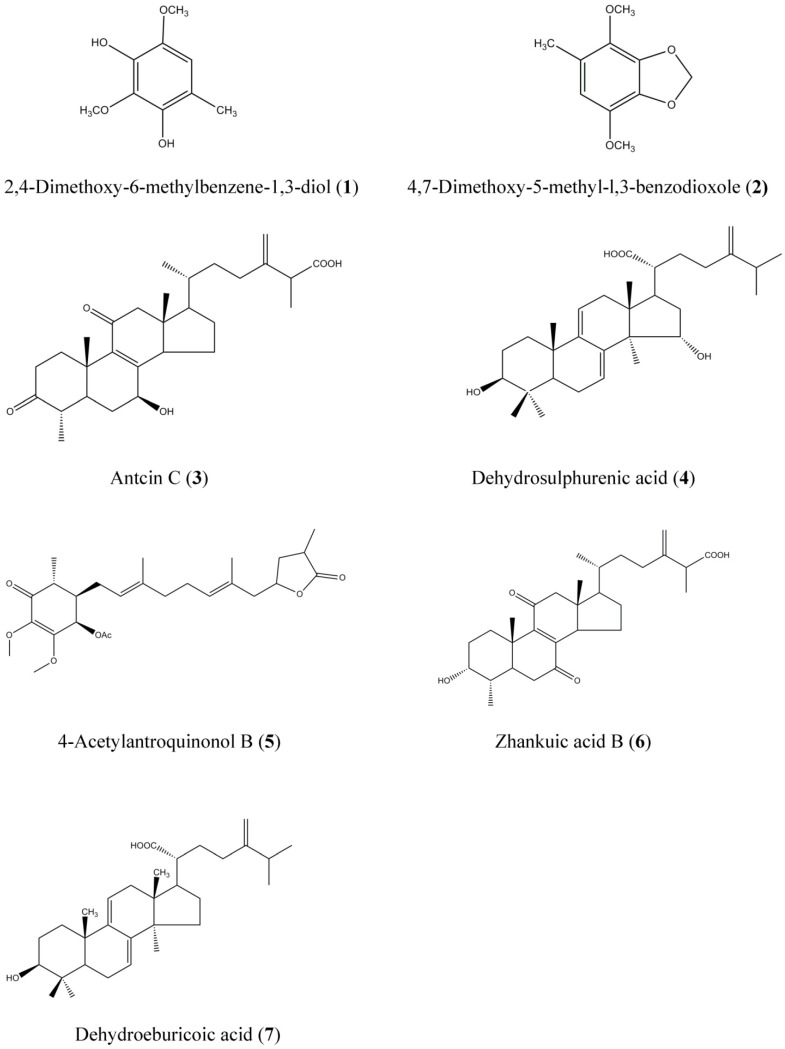
Chemical structures of triterpenoid and benzenoid compounds from solid-cultured mycelia of *A. cinnamomea*.

Supercritical fluids potentially have highly useful physical properties, such as low viscosity and high diffusivity into the sample matrix. Carbon dioxide (CO_2_) is the most widely used solvent among the supercritical fluids due to its harmless, safe, non-explosive, readily available characteristics and has been classified as Generally Recognized as Safe (GRAS) by the Food and Drug Administration (FDA). However, the disadvantage of SF-CO_2_ is its limited extractability of compounds with hydrophilic character from natural sources [16]. Addition of an organic modifier results in a greatly improved extraction efficiency and efficiently extracting active compounds of diverse polarity without the limitations for lipophilic compounds [16]. This trend has been proved in the extraction of polyunsaturated fatty acids, including eicosapentaenoic acid (EPA), from the filamentous fungus *Saprolegnia parasitica*. It was indicated that the recovery of lipid increases with increasing pressures and higher recoveries were obtained when a mixture of CO_2_ with 10% (w/w) ethanol was used as the co-solvent [17]. As mentioned in the Experimental Section, the extractions obtained with SFE under diverse extraction conditions of temperature (T) and pressure (P) and one classical solvent extraction using ethanol were compared for extraction efficiencies in terms of yield and total triterpenoid content in AC. The highest yield (18.38% ± 0.52%, w/w) in the overall SFE was obtained using the optimal conditions of 34.5 Mpa (5,000 psi)/60 °C (S-5000-60). Although the extraction yield of S-5000-60 was less than that of ethanolic extraction (EtOH-E, 32.26% ± 1.34%) (Table 1), however, the total triterpenoid content in the two samples was nearly equivalent (59.70 ± 6.04 *vs.* 60.33 ± 0.04 mg/g). Moreover, a higher triterpenoid percentage was obtained from S-5000-60 (1.68%) when compared to the extract obtained by the conventional ethanol solvent extraction method (1.28%). We therefore recommend this sample for further studies on the subsequent purification on the main compounds from sample S-5000-60 by semipreparative HPLC that led to the isolation of seven compounds, as shown in Figure 1. These compounds were identified as 2,4-dimethoxy-6-methylbenzene-1,3-diol (**1**), 4,7-dimethoxy-5-methyl-l,3-benzodioxole (**2**), antcin C (**3**), dehydrosulphurenic acid (**4**), 4-acetylantroquinonol B (**5**), zhankuic acid B (**6**) and dehydroeburicoic acid (**7**) by H^1^-NMR and ESI-MS-MS data and this was confirmed by comparison with those reported in the literature [18,19,20,21,22,23,24].

**Table 1:** Yield, total triterpenoids, contents in *A. cinnamomea* solid culture extracts obtained under different supercritical fluid and ethanol solvent extraction conditions.

Extract ^1^	Yeild (%)	Total Triterpenoids ^2^	Content (mg/g Extract, DW) ^3^						
			1	2	3	4	5	6	7
S-3000-40 ^1^	11.83 ± 0.75 ^de^	22.83 ± 3.99 ^e^	2.95 ± 0.02 ^d^	5.88 ± 0.34 ^e^	0.10 ± 0.01 ^bcd^	0.02 ± 0.006 ^d^	0.21 ± 0.03 ^bc^	0.01 ± 0.004 ^b^	0.11 ± 0.01 ^de^
S-3000-60 ^1^	13.15 ± 0.64 ^d^	40.17 ± 1.94 ^d^	3.86 ± 0.31 ^c^	6.58 ± 0.25 ^cd^	0.12 ± 0.03 ^bc^	0.05 ± 0.004 ^c^	0.23 ± 0.05 ^bc^	ND ^4^	0.98 ± 0.02 ^a^
S-3000-80 ^1^	10.43 ± 0.90 ^e^	21.31 ± 2.50 ^cd^	3.86 ± 0.09 ^c^	6.23 ± 0.07 ^d^	0.07 ± 0.01 ^cd^	0.04 ± 0.001 ^cd^	0.20 ± 0.07 ^bc^	ND	0.07 ± 0.04 ^e^
S-4000-40 ^1^	15.76 ± 0.46 ^c^	33.48 ± 3.61 ^cd^	3.96 ± 0.05 ^c^	7.98 ± 0.12 ^b^	0.18 ± 0.02 ^b^	0.02 ± 0.007 ^d^	0.29 ± 0.01 ^ab^	0.002 ± 0.0002 ^c^	0.19 ± 0.01 ^d^
S-4000-60 ^1^	16.09 ± 1.22 ^c^	49.88 ± 2.88 ^b^	4.75 ± 0.53 ^b^	8.69 ± 0.34 ^a^	0.31 ± 0.31 ^a^	0.04 ± 0.003 ^cd^	0.34 ± 0.08 ^a^	0.03 ± 0.001 ^a^	0.34 ± 0.08 ^c^
S-4000-80 ^1^	16.97 ± 1.47 ^bc^	42.12 ± 5.06 ^a^	4.20 ± 0.34 ^bc^	6.89 ± 0.34 ^c^	0.34 ± 0.12 ^a^	0.12 ± 0.004 ^b^	0.26 ± 0.05 ^ab^	ND	0.37 ± 0.11 ^bc^
S-5000-40 ^1^	16.40 ± 1.06 ^bc^	35.39 ± 4.02 ^e^	3.86 ± 0.13 ^c^	7.70 ± 0.21 ^b^	0.16 ± 0.01 ^bc^	0.02 ± 0.001 ^d^	0.27 ± 0.06 ^ab^	0.03 ± 0.002 ^a^	0.17 ± 0.03 ^de^
S-5000-60 ^1^	18.38 ± 0.52 ^b^	59.70 ± 0.40 ^a^	3.03 ± 0.05 ^d^	5.78 ± 0.02 ^e^	0.37 ± 0.04 ^a^	0.12 ± 0.03 ^b^	0.23 ± 0.02 ^bc^	0.004 ± 0.0003 ^c^	0.40 ± 0.02 ^bc^
S-5000-80 ^1^	15.41 ± 2.24 ^c^	40.18 ± 6.24 ^cd^	3.34 ± 0.07 ^d^	6.11 ± 0.15 ^e^	0.28 ± 0.05 ^a^	0.10 ± 0.02 ^b^	0.16 ± 0.01 ^c^	ND	0.31 ± 0.12 ^c^
Ethanol	32.26 ± 1.34 ^a^	60.33 ± 0.04 ^a^	6.98 ± 0.15 ^a^	8.13 ± 0.07 ^b^	0.01 ± 0.003 ^d^	0.32 ± 0.03 ^a^	0.29 ± 0.06 ^ab^	0.01 ± 0.002 ^b^	0.46 ± 0.06 ^b^

^1^ S-3000-40, 60 and 80; S-4000-40, 60 and 80; S-5000-40, 60 and 80: SFE extract at 20.7, 27.6 and 34.5 MPa (corresponding to 3000, 4000 and 5000 psi, respectively) and 40, 60, and 80 °C, respectively; ^2^total triterpenoids content of extracts was expressed as mg ursolic acid equivalents (GAE)/g extract; ^3^ data are referred to the calibrated standard curves of related isolated and purified compounds from AC mycelia by HPLC analysis ; ^4^ not detected; ^a b c d e^ mean values within a column with dissimilar superscript letters are significantly different (*p* < 0.05).

### 2.2. HPLC and LC-MS/MS Analysis of AC Extracts

**Figure 2:**
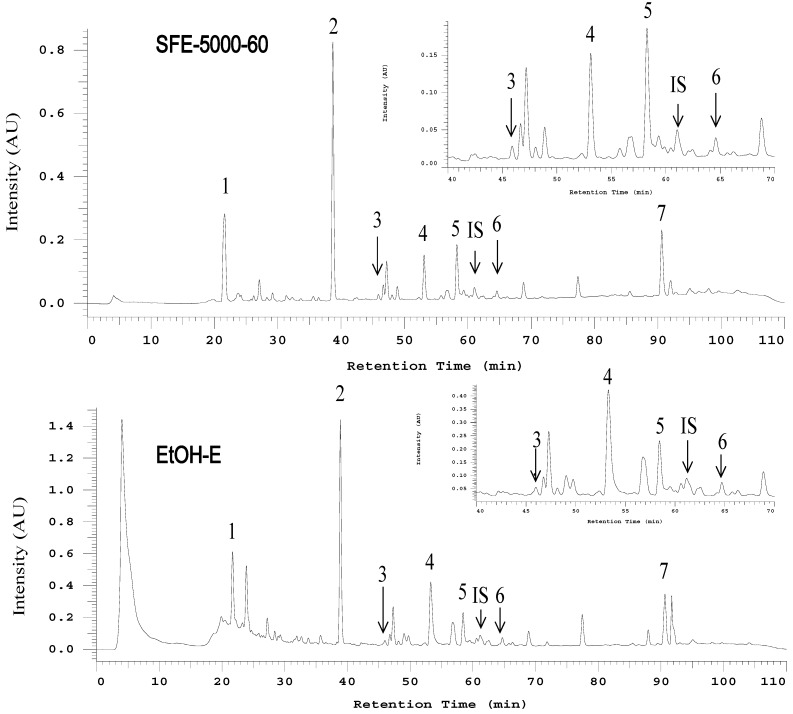
.The HPLC-UV chromatograms (detected at 254 nm) of S-5000-60 and EtOH-E extracts of solid cultured mycelial *Antrodia cinnamomea.* Peak numbers are referred to Table 1.

Comprehensive HPLC profiles of AC mycelia prepared from the SFE at 34.5 MPa (5,000 psi)/60 °C (S-5000-60) and ethanolic extract were established as shown in Figure 2. Under 254 nm wavelength detection, seven major peaks matching the structures shown in Figure 1 were labeled in the chromatograms of the examined samples and applied for the isolation and purification by preparative HPLC.

**Figure 3:**
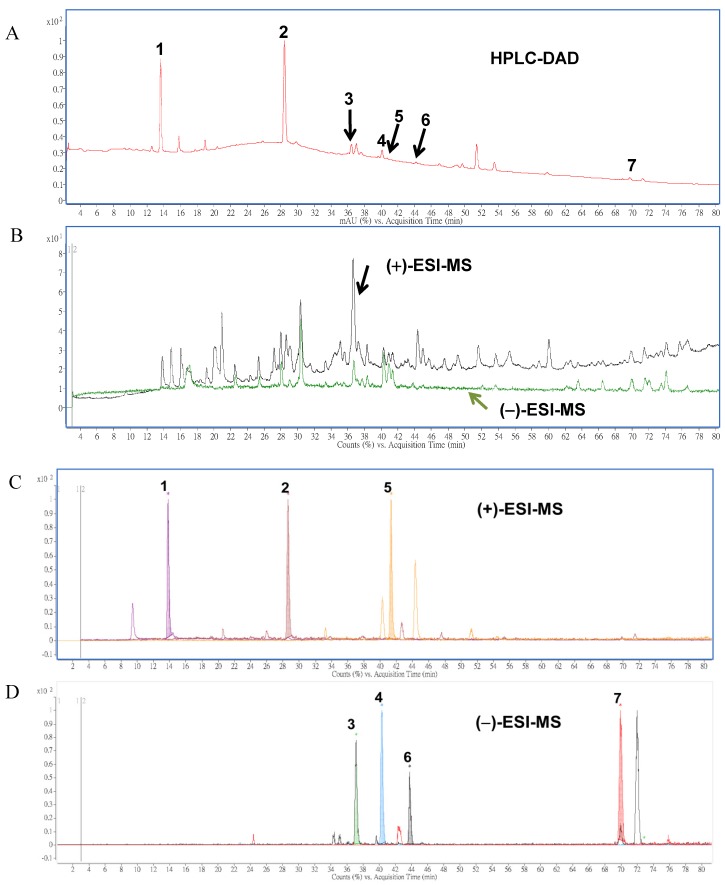
HPLC (**A**) and total ion chromatograms (TIC) of ESI-MS in positive (black line) and negative (green line) modes; (**B**) and the overlaid chromatograms of the molecular ions of benzenoid; (**C**) and triterpenoid compounds; (**D**) obtained by positive and negative ESI-MS analysis, respectively. Peak numbers and compound names correspond with Figure 1.

To establish the index compounds in solid-cultured mycelia of AC, HPLC-DAD-ESI-MS/MS was employed for further identification of the constituents in AC extracts. The HPLC analysis indicated less detected peaks shown in the chromatogram (Figure 3A) than that of ESI-MS/MS techniques in both positive and negative ionization modes used in the identification (Figure 3B). Data obtained from the positive or negative ESI-MS and ESI-MS-MS analyses of the seven compounds are summarized and shown in Table 2.

**Table 2:** Chemical characterization of the main compounds detected in the supercritical fluid and ethanolic extracts of solid-cultured mycelia of *A. cinnamomea* by liquid chromatography-tandem mass spectrometry (LC-MS/MS) analysis.

Peak No.	Compounds ^a^	*t*_R_ (min)	UV-Vis Absorbance (nm)	[M + H]^+^	[M − H]^−^	MS-MS Major Fragments
1	2,4-Dimethoxy-6-methylbenzene-1,3-diol	13.6	286, 236	185	- ^b^	153, 137, 170
2	4,7-Dimethoxy-5-methyl-l,3-benzodioxole	25.8	236, 284, 212(sh)	197	-	139, 167, 182, 137
3	Antcin C	37.0	255, 288(sh)	-	469	425, 341, 407, 301, 247
4	Dehydrosulphurenic acid	40.1	266, 233	485	-	471, 389
5	4-Acetylantroquinonol B	40.7	264, 232(sh)	463	-	403, 420, 389, 235
6	Zhankuic acid B	44.2	256, 233(sh)	-	469	425, 407
7	Dehydroeburicoic acid	69.8	246, 254(sh), 231(sh)	469	-	311, 451

^a^ Identification was compared with those of identified compounds from literatures [18,19,20,21,22,23,24]; ^b^ Not identified.

**Table 3:** Calibration curves of three benzenoids and four triterpenoids in solid-state cultured mycelia of *Antrodia cinnamomea* determined by HPLC-UV.

Peak No.	Compound Name	Linearity Range (µg/mL)	Equation	R^2^
1	2,4-Dimethoxy-6-methylbenzene-1,3-diol	10~600	y = 0.076x + 0.5363	0.9986
2	4,7-Dimethoxy-5-methyl-1,3-benzodioxole	10~800	y = 0.0821x − 0.0556	0.9955
3	Antcin C	0.1~20	y = 0.4367x + 0.6518	0.9952
4	Dehydrosulphurenic acid	0.1~50	y = 0.6956x + 0.7382	0.9953
5	4-Acetlyantroquinonol B	0.1~50	y = 0.5478x − 0.0415	0.9921
6	Zhankuic acid B	0.1~20	y = 0.9429x − 0.0199	0.9967
7	Dehydroeburicoic acid	1~50	y = 0.3893x + 0.8817	0.993

It was found that the positive mode resulted in a larger number of ions compared to the negative mode (Figure 3B). However, after the comparison of main peaks intensity for molecular ions of (+)- and (−)-ESI-MS, we found that peaks **1**, **2** and **5** were significantly present in the total ion chromatogram (TIC) after screening of the molecular ions of each compound in positive ionization mode (Figure 3B, upper panel), while peaks **3**, **4**, **6**, and **7** were detected with high intensity in negative ionization mode (Figure 3B, lower panel). This significant difference led to the conclusion that the negative ESI-MS mode was more suitable for the analysis of triterpenoid compounds (such as **3**, **4**, **6**, and **7**), while the analysis of benzenoid compounds (**1**, **2**, and **5**) using positive mode would be more appropriate.

To measure the content of each mentioned compound in both of extracts, calibration curves of the seven compounds were established using seven dilution standards from 0.1 to 800 μg/mL. Table 3 shows the regression parameters and linearity of the proposed HPLC profiling method on the three major benzenoid and four triterpenoid compounds. Content of the seven major constituents (Table 1) was determined by using the calibration curves of index compounds, respectively and the Equation (1) as described in the Experimental Section. As shown in Table 1, compound **2** (5.78 mg/g) was the most abundant one in the mycelium extract (S-5000-60) of solid-state culture of AC, followed by compound **1** (3.03 mg/g). In a previous report [19], compound **2** had indicated the capability to decrease the proliferation of human colon cancer cells (COLO 205) through G0/G1 cell-cycle arrest and induction of apoptosis. In addition, the second highest amount of compound **1** has been isolated from AC fruiting body and demonstrated bioactivity by decreasing the growth of COLO-205 human colon cancer cell tumor xenografts in an athymic nude mouse model [25]. These results were quite comparable with those contents determined for ETOH-E. Surprisingly, antcin C (**3**) was found to be significantly enriched (0.37 ± 0.04 mg/g) in S-5000-60, 30 times more than the level of that compound in EtOH-E (0.01 ± 0.003 mg/g). Antcin C, a kind of zhankuic acid derivative, has indicated significant anti-proliferative effects at a concentration of 28.82 µg/mL against human acute lymphoblastic leukemia cells [26]. The specific cause was supposed to be due to the functional group at the C3 position. If a hydroxyl group exists at the C-3 position the compound will present less cytotoxic activity, while a compound with a carbonyl group in that position will have a significant cell proliferation inhibition activity [26]. Therefore, the other three triterpenoid compounds **4**, **6** and **7**, which have hydroxyl groups at the C3 position, could be supposed to have non-equivalent potency like compound **3**.

### 2.3. Inhibitory Effect on Proliferation of HepG2 Cells

**Figure 4:**
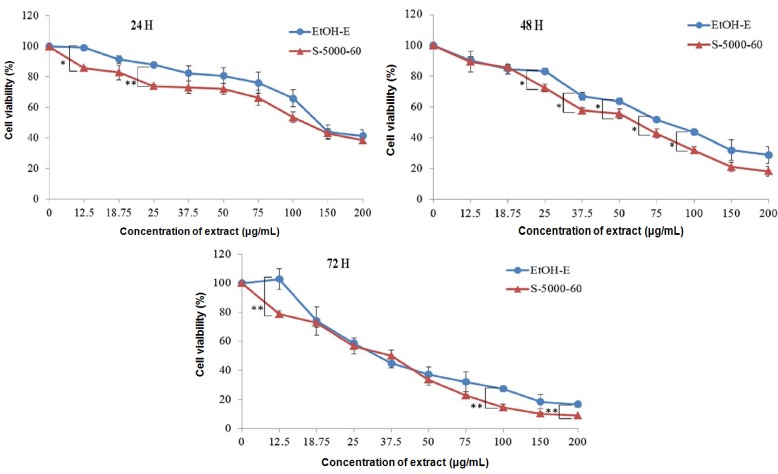
Effects of S-5000-60 and EtOH-E extracts on hepatocellular carcinoma (HepG2) cells proliferation at 24, 48 and 72 h treatment, respectively. Cells were treated with varying concentrations of S-5000-60 and EtOH-E. The growth inhibition rate of HepG2 cells was assessed by 3-(4,5-dimethylthiazol-2-yl)-2,5-diphenyltetrazolium bromide assay. * *p* < 0.05 *vs.* Control; ** *p* < 0.01.

All SFEs and EtOH-E were subjected to a cytotoxicity assay against HepG2 (Figure 4) by an MTT method and the IC_25_ and IC_50_ values (Table 4) were derived from the corresponding dose-response curves (Figure 4) for treatments of 24, 48 and 72 h, respectively. A 24 h exposure to S-5000-60 decreased the proliferation of HepG2 cells in a concentration-responsive manner with an IC_50_ at 116.15 μg/mL, a higher inhibition effect than the EtOH-E sample (131.09 μg/mL). Furthermore, IC_50_ values were decreased to 43.96 and 48.30, respectively in S-5000-60 and EtOH-E after the exposure times up to 72 h. This result indicates that S-5000-60 may possess a relatively more effective cytotoxicity toward hepatoma cancer cells than EtOH-E.

**Table 4:** Anti-proliferation effects of supercritical fluid and ethanolic extracts of solid-cultured mycelia of *A. cinnamomea* on human hepatocellular carcinoma HepG2 cells.

Treatment	Time (hour)					
Inhibition	24 h		48 h		72 h	
Concentration ^1^	EtOH-E ^2^	S-5000-60 ^3^	EtOH-E	S-5000-60	EtOH-E	S-5000-60
IC_25_	77.88 ^a^	41.15 ^b^	25.67 ^bc^	26.40 ^bc^	18.47 ^c^	14.85 ^c^
IC_50_	131.09 ^a^	116.15 ^b^	80.04 ^c^	57.82 ^c^	48.30 ^d^	43.96 ^d^

^1^ Each value of inhibition concentration (g/mL) represents mean ± SD; ^2^ EtOH-E: ethanolic extract; ^3^ S-5000-60: supercritical fluid extract obtained at 34.5 MPa (5000 psi) and 60 °C; ^a b c d ^mean values within a row with dissimilar superscript letters are significantly different (*p* < 0.05).

### 2.4. The Apoptotic Activities of S-5000-60 and EtOH-E Extracts

**Figure 5:**
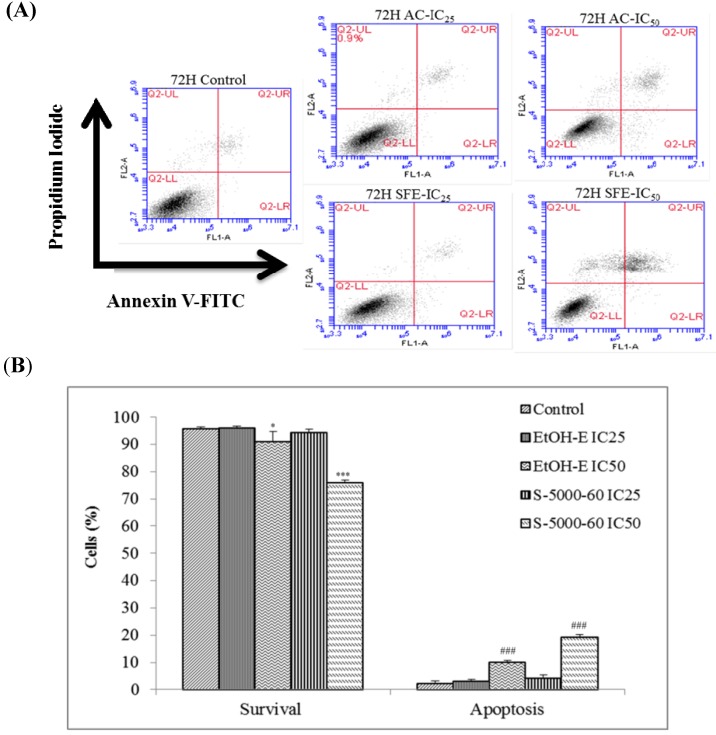
The effects of S-5000-60 and EtOH-E extracts on apoptosis induction in HepG2 cell. Representative dot-plots (**A**) and the percentages of apoptosis in HepG2 cells; (**B**) from three independent experiments for the quantitative assessment of apoptosis induction by using Annexin V and PI staining after treatment with extracts at 72 h. The total percentage of viable cells, cells undergoing apoptosis, apoptotic cells, and necrosis cells is taken as 100%. Values are the mean ± SD of three independent experiments. * *p* < 0.05 *vs.* Control; *** *p* < 0.001; ^###^ *p* < 0.001.

To further compare the cytotoxic activity of the S-5000-60 and EtOH-E extracts, apoptotic cells were stained with annexin V-FITC and propidium iodide (PI) and measured by using a flow cytometer on logarithmic scales and analyzed as bivariate dot-plots. As the representative result shown in Figure 5 indicates, apoptosis ratios (including the early and late apoptosis ratios) for S-5000-60 and EtOH-E obtained after 72 h treatment at concentrations of IC_25_ and IC_50_ doses, respectively, resulted in a highest apoptosis ratio being 21.5% from S-5000-60 at the IC_50_ dose, significantly (*p* < 0.05%) higher than the 10.5% ratio of ETOH-E.

## 3. Experimental

### 3.1. Chemicals, Reagents and Antibodies

Trypan Blue, 3-(4,5-dimethylthiazol-2-yl)-2,5-diphenyltetrazolium bromide (MTT), dimethyl sulfoxide, (DMSO), formic acid and mouse monoclonal antibody specific for β-actin were provided by Sigma-Aldrich (St. Louis, MO, USA). Acetonitrile, glacial acetic acid, fetal bovine serum (FBS), L-glutamine solution (100 mM), penicillin-streptomycin (5,000 units/mL penicillin and 5 mg/mL) were purchased from Biological Industries (Beit Haemek, Israel). Dulbecco’s Modified Eagle Medium (DMEM) and trypsin-EDTA solution were provided by Hyclone (Logan, UT, USA). FITC Annexin V/PI apoptosis detection kit was purchased from BD Biosciences (San Diego, CA, USA).

### 3.2. Solid-State Culture of A. cinnamomea

The BCRC 36795 strain of *A. cinnamomea* was purchased from the Food Industry Research and Development Institute (FIRDI, Hsinchu, Taiwan). The fungus was maintained on potato dextrose agar plate at 25 °C. Then, 10 day-old culture was inoculated into a culture medium that was composed of 25 mL of 0.5% yeast extract and 25 g of buckwheat powder in a glass bottle at 27–30 °C for 60 days under dark light. Fungal mycelium was then harvested, freeze-dried, weighted, and stored at −20 °C for further studies.

### 3.3. Supercritical Fluid and Ethanol Solvent Extraction of A. cinnamomea Mycelia

Supercritical fluid extraction (SFE) was used to extract triterpenoids and benzenoids from AC according to the report of Kuo *et al.* [27] with some modifications. In brief, freeze-dried and pulverized culture product (5.0 g) was placed in the extraction vessel (10 mL) of the supercritical fluid extraction apparatus (Model SFX 2-10, ISCO, Lincoln, NE, USA). Two ISCO Model 260D syringe pumps were used for pumping solvent (carbon dioxide, 99.95% purity) and co-solvent (95% ethanol). Extraction conditions were operated independently at 20.7, 27.6 and 34.5 MPa (corresponding to 3,000, 4,000 and 5,000 psi, respectively) with 10% co-solvent in combination with temperatures at 40, 60, and 80 °C, respectively, for 30 min of static and followed by another 60 min of dynamic extractions in a solvent flow rate of 1.0 mL/min. Extracted constituents were collected in a 20 mL vial that was prefilled with a trapping solvent (10 mL of ethanol) and maintained at 4 °C during the extraction step. The extracted sample of SFE was evaporated to dryness in a Büchi rotatory evaporator under reduced pressure at 40 °C.

Conventional solvent extraction with ethanol was also conducted for comparative evaluation in the study. In brief, the dried and pulverized culture product was extracted thrice with 95% ethanol in a ratio of 1:10 (w/v) at room temperature for 8 h each under constant stirring. The ethanol solution was concentrated to dryness *in vacuo.* Each extraction was completed in triplicate, and the extraction recoveries reported are the average value of three extractions.

### 3.4. Determination of Total Triterpenoids

According to Fan and He [28], the content of the total triterpenoids was determined by the vanillin/glacial acetic acid method. Briefly, each extract obtained as described in Section 3.3 was dissolved in methanol in a concentration of 1 mg/mL, respectively. One hundred µL of methanol solution was then added into the tube, followed successively by 150 µL of 5% vanillin/glacial acetic acid (w/v) and 500 µL of perchloric acid solution, and the sample solution was heated for 45 min at 60 °C and then cooled in an ice-water bath to ambient temperature. The absorbance of the sample was measured at 548 nm after being diluted to 2.25 mL with glacial acetic acid. A calibration curve was established using ursolic acid as the reference compound, from which the total triterpenoid content was estimated. 

### 3.5. HPLC and LC-MS/MS Analysis of Extracts

#### 3.5.1. Preparation of Calibration Standards

Each of the standard solutions of the purified compounds from S-5000-60 of AC was first prepared in ethanol at a concentration of 100 mg/L and was gradually diluted in mobile phase to working concentrations of 0.1 to 50 mg/L. To each standard solution, glycyrrhetinic acid (Sigma-Aldrich) was added to make-up a final IS concentration of 1.0 μg/mL. The calibration curves were constructed with six points, in triplicate, for each purified compound, using the internal standard method.

#### 3.5.2. Quantification of the Major Components in Extracts

Quantitative analysis of the nine SFE extracts and EtOH-E was performed on a Hitachi (Model L2130, Tokyo, Japan) liquid chromatograph system, equipped with a vacuum degasser, quaternary pump (Chromaster 5110), diode-array detector (L-7455) and an autosampler (Chromaster 5210) with a 20 μL loop. A Luna C18(2) reversed-phase analysis column (2.0 mm × 150 mm, 3 μm particle size) in series connected to a precolumn (Security Guard C18(ODS) 4 mm × 3.0 mm ID, Phenomenex Inc., Torrance, CA, USA) was used for the separation of components in extracts. Gradient elution using solvent A (water, containing 0.1% formic acid, FA) and solvent B (acetonitrile, containing 0.1% FA) as the mobile phase at a flow rate of 0.2 mL/min was applied. The gradient conditions were: 0–3 min, 10% B, 3–10 min, linear from 10% to 35% B, 10–25 min linear from 35% to 50% B; 25–40 min linear from 50% to 60% B, 40–75 min linear from 60% to 85% B, 75–80 min linear from 85% to 95% B, 80–90 min, isocratic at 95% B then returned to 10% B in 10 min. Peak areas were determined at 254 nm for the main seven compounds present in AC extracts and the quantification were quantified regarding each pure standard curve. Each content of the main seven compounds in AC was quantified using a formula reported by Chen *et al.* [29] as given below:

triterpenoids or benzenoids (µg/g) = 
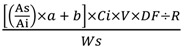
(1)
wherein, As: peak area of tritepenoid acid or benzenoid; Ai: peak area of internal standard; *a*: slope of calibration curve; *b*: intercept of calibration curve; *Ci*: concentration of internal standard; *V*: volume of extract; *DF*: dilution factor; *R*: recovery; *Ws*: weight of sample (g).

#### 3.5.3. LC-Tandem Mass Spectrometry

The same column used as above was placed in an Agilent 6420 Triple Quadrupole Mass Spectrometer equipped with Mass Hunter software (version: B.01.04; Agilent Technologies, Santa Clara, CA, USA). The gradient conditions were: 0–3 min, isocratic at 10% B; 3–15 min: 10%–50% B; 15–40 min: 50%–70% B; 40–60 min: 70%–95% B; 60–75 min held at 95%–5% B then returned to 10% B in 5 min. The system was operated in electrospray ionization (ESI) with positive and negative ionization modes. Nitrogen was used both as a drying gas at a flow rate of 9 L/min and as nebulising gas at a pressure of 40 psi. The drying gas temperature was 325 °C, and a potential of 3700 in positive or −3500 V in negative ionization was applied across the capillary. The fragmentor voltage was 110 V, and the collision voltage was 15 V. Quadrupole 1 filtered the calculated *m*/*z* of each compound of interest, whilst quadrupole 2 scanned for ions produced by nitrogen collision of these ionized compounds in the range 100–800 *m*/*z* at a scan time of 200 ms/cycle. The identification of benzenoid and triterpenoid compounds was obtained by matching their molecular ions (*m*/*z*) obtained by ES positive and negative ionization with those references of isolated compounds.

### 3.6. Effects of Extracts on the Proliferation of HumanHepatocellular Carcinoma HepG2 Cells

#### 3.6.1. Cell Culture and Cell Viability Assay

Cultivation of HepG2 cells and the MTT assay were performed as previously reported [30]. Human hepatoma HepG2 cells, obtained from the Bioresource Collection and Research Center (BCRC, Hsinchu, Taiwan), were cultured in Dulbecco’s modified Eagle’s medium (DMEM) supplemented with 10% fetal bovine serum (FBS), 100 U/mL of penicillin, 0.1 g/L of streptomycin and 4 mM glutamine, under 95% air and 5% CO_2_ at 37 °C. SFE-E and EtOH-E extracts were dissolved in DMSO to form 10 mg/mL stock solution and stored at −80 °C until used, and then added to the culture medium at a final concentration of 25, 75, 150, and 200 μg/mL to perform the *in vitro* cytotoxicity and other relevant experiments. DMSO at 1% (v/v), which did not significantly affect the assays described below, served as the solvent control. Cells were seeded onto a 96-well plate at a density of 3000 cells/well. After 24 h the culture medium was replaced with SFE or EtOH extract solutions at different dilutions as indicated. The incubation was further continued for additional 24, 48 and 72 h, respectively, and the MTT colorimetric assay was conducted following the manufacturer’s instruction. To each well 50 µL of MTT solution (5 mg/mL in double distilled water) was added to yield a final concentration of 0.5 mg/mL. After incubation for 4 h, the unreacted dye was removed by aspiration. To dissolve the purple formazan product, 100 µL/well of DMSO was added. The absorbance was taken at 570 nm using a microplate reader (Multiskan Spectrum, Thermo Electron Corporation, Waltham, MA, USA).

#### 3.6.2. Apoptosis Assay by Annexin V FITC/PI Double-Staining

Cells (5.5 × 10^6^/mL) were cultured in six-well plates for 24 h and then treated with S-5000-60 and EtOH-E at IC_25_ and IC_50_ doses, respectively for 72 h. Cells without extracts added were used as the control. Apoptosis was detected according to the Annexin V-FITC Apoptosis Detection kit (BD Biosciences) and with slight modification from the previous study [31]. Cells were centrifuged and resuspended in binding buffer at a ratio of 1 × 10^5^/mL cells/mL. The suspensions were transferred to 5 mL tubes and 5 µL Annexin V-FITC (BD Biosciences) plus 5 µg/mL PI (BD Biosciences) were added. The cells were incubated at 4 °C for 30 min and after the addition of 0.3 mL of binding buffer, the analysis was performed in BD Accuri C6 flow cytometer (BD Biosciences, Ann Arbor, MI, USA) with the as-built software for the data processing (10,000 events were collected per sample). Experimental controls were performed with cells treated only with the medium. Data were presented as media ± SD of the triplicates. The test has been used to discriminate intact cells (FITC^−^/PI^−^), apoptotic cells (FITC^+^/PI^−^) and necrotic cells (FITC^+^/PI^+^) [32].

### 3.7. Statistical Analysis

Data obtained were statistically treated with a one-way analysis of variance (ANOVA). Tukey’s test or a least significant different (LSD) test was used to analyze differences in significance. *p* < 0.05 was considered to indicate a significant difference between groups.

## 4. Conclusions

*Antrodia cinnamomea* solid-state cultured mycelia extracts obtained by SFE (S-5000-60) showed lower yield but a higher percentage of triterpenoids when compared to the extract obtained by the conventional ethanol solvent extraction method. The SFE method used for the extraction of triterpenoids and benzenoids proved to be more promising than ethanol solvent extraction for more specific extraction on the target compounds. Cytotoxicity of the extracts against HepG2 cells indicated the significantly higher activity of S-5000-60, which was up to two times more effective than EtOH-E according to the Annexin V FITC/PI double-staining assay. Among the identified compounds in both extracts, antcin C was identified as the most potent candidate in inducing the apoptosis of HepG2 cells through the comparison of chemical structure differences in triterpenoids and the content of triterpenoid compounds between S-5000-60 and EtOH-E extracts.
